# Bio-Inspired Network for Diagnosing Liver Steatosis in Ultrasound Images

**DOI:** 10.3390/bioengineering10070768

**Published:** 2023-06-26

**Authors:** Yuan Yao, Zhenguang Zhang, Bo Peng, Jin Tang

**Affiliations:** 1 General Practice Medical Center, West China Hospital, Sichuan University, Chengdu 610044, China; yaoyuan@wchscu.cn; 2 School of Automation, Guangxi University of Science and Technology, Liuzhou 545006, China; 3 School of Computing and Artificial Intelligent, Southwest Jiaotong University, Chengdu 611756, China; bpeng@swjtu.edu.cn; 4 Tiaodenghe Community Health Service Center, Chengdu 610066, China; tangjin0120@126.com

**Keywords:** fatty liver ultrasound images, liver steatosis, biological vision, self-attention, transformer

## Abstract

Using ultrasound imaging to diagnose liver steatosis is of great significance for preventing diseases such as cirrhosis and liver cancer. Accurate diagnosis under conditions of low quality, noise and poor resolutions is still a challenging task. Physiological studies have shown that the visual cortex of the biological visual system has selective attention neural mechanisms and feedback regulation of high features to low features. When processing visual information, these cortical regions selectively focus on more sensitive information and ignore unimportant details, which can effectively extract important features from visual information. Inspired by this, we propose a new diagnostic network for hepatic steatosis. In order to simulate the selection mechanism and feedback regulation of the visual cortex in the ventral pathway, it consists of a receptive field feature extraction module, parallel attention module and feedback connection. The receptive field feature extraction module corresponds to the inhibition of the non-classical receptive field of V1 neurons on the classical receptive field. It processes the input image to suppress the unimportant background texture. Two types of attention are adopted in the parallel attention module to process the same visual information and extract different important features for fusion, which improves the overall performance of the model. In addition, we construct a new dataset of fatty liver ultrasound images and validate the proposed model on this dataset. The experimental results show that the network has good performance in terms of sensitivity, specificity and accuracy for the diagnosis of fatty liver disease.

## 1. Introduction

Fatty liver disease (the abnormal accumulation of fat in hepatocytes exceeding 5%) is generally considered to be the main cause of liver diseases such as cirrhosis, liver cancer, liver failure, etc. [1,2]. Therefore, the diagnosis and classification of fatty liver have practical significance for the prevention of such diseases and human health. Currently, ultrasound is widely used for the diagnosis of fatty liver due to its advantages of being non-invasive, low-cost, and wide availability. Clinicians evaluate the images by observing features such as enhanced liver and kidney echogenicity, blurred portal or hepatic vein vessels, and bright liver echogenicity in ultrasound images [3]. However, the low quality of ultrasound images, containing speckle noise and blur, and the subjective nature of the assessment (susceptible to clinician experience and ultrasound acquisition equipment settings [4,5]) have led to a certain degree of misdiagnosis [6,7]. In addition, several studies have shown that the sensitivity of ultrasound diagnosis is 93% when steatosis exceeds 30%, and if steatosis is less than 20%, the specificity and sensitivity of ultrasound images are poor [3]. Therefore, using ultrasound imaging to diagnose liver steatosis has always been a challenging visual task. How to design high-performance and high-accuracy classification models is still an urgent problem to solve.

To achieve better performance in fatty liver ultrasound image classification, some researchers have improved the quality of ultrasound images by reducing speckle noise, which enhances the accuracy of the model’s classification. In addition, earlier research methods have been used to diagnose liver steatosis levels more accurately by applying complex algorithms [8], statistical models [9], image processing techniques [10], or traditional machine learning methods [11], such as liver and kidney index (HRI), gray-level co-occurrence matrix (GLCM) [12,13], and machine learning methods (support vector machines and K-nearest neighbors, etc.). These efforts have improved the accuracy of fatty liver diagnosis to some extent. However, early research methods still have some problems and limitations, such as the potential to blur images when reducing speckle noise, the need to rely on skill in selecting regions of interest (ROI), the subjective experience of clinicians when diagnosing using complex algorithms or image processing techniques, the need to manually design features when utilizing traditional machine learning methods, and the inability of such features to be optimized as the data set changes. In recent years, with the wide applications of CNN in the field of computer vision, more and more scholars have proposed liver ultrasound image classification methods based on CNN, and obtained good performance. For example, Zhang et al. [14] used a shallow CNN-based model to extract texture features from ultrasound images and detect the level of liver steatosis. Reddy et al. [15] trained and tested the proposed CNN method using 48 × 48 texture patches and achieved an accuracy of 93.5%. Biswas et al. [16] proposed a two-class CNN architecture for fatty liver disease classification. It achieved a 100% classification accuracy by evaluating ultrasound images of 63 patients (27 normal/36 abnormal) under tenfold cross-validation conditions. Later, Byra et al. [17] used CNN models trained on other tasks for fatty liver ultrasound image classification by transfer learning, and compared the results with those of HRI and GLCM. The results showed that CNN pre-trained on other tasks produced better results. In addition, Kuppili et al. [18] proposed an extreme learning machine (ELM)-based fatty liver classification method with an average classification accuracy of 92.4%. Meng et al. [19] proposed a fully connected neural network (FCNet) to achieve liver fibrosis classification by training and testing using regions of interest with an accuracy of 63.24%.

**Figure 1:**
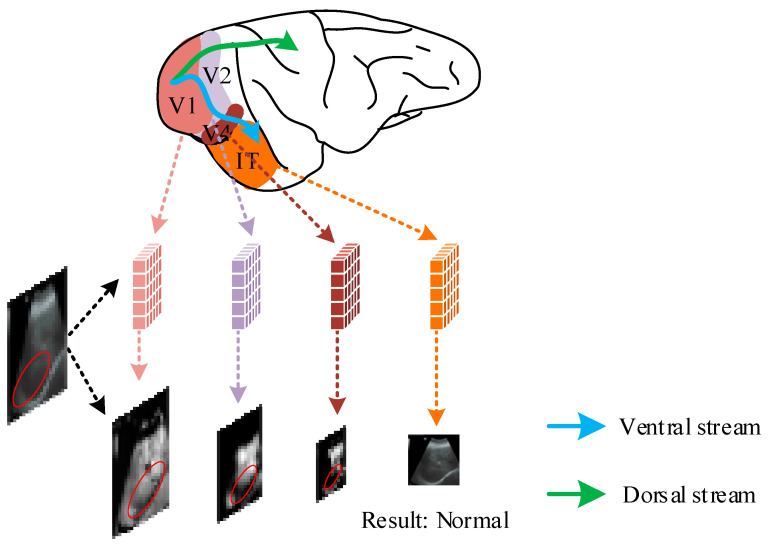
Processing and transmission of visual information in ventral pathway. Among them, the blue arrow indicates the processing and transmission direction of visual information in the ventral channel, and the green arrow indicates the processing and transmission direction of the dorsal channel.

Compared with earlier research methods, the convolutional neural network-based liver ultrasound classification method obtains better classification performance by extracting texture features in images, grayscale features, and fusing feature information at different scales, and does not require extensive hand-designed features and subjective experience. However, as deep learning techniques continue to evolve, some researchers have found it difficult to achieve performance breakthroughs with models based solely on experience and experiments. For this reason, some researchers [20,21,22] have proposed new bionic models inspired by the biological vision mechanism, and have achieved good performance in various visual tasks. For example, Grigorescu et al. [23] proposed a contour detection model to suppress the image background texture based on the inhibitory effect of the non-classical receptive field (nCRF) response on classical receptive field (CRF)response. Yang et al. [24] combined the color antagonism mechanism in the visual pathway with the spatial sparseness strategy (SSC), and proposed an Color-Opponency and Spatial Sparseness Constraint (SCO) model for edge detection. Later, inspired by them, some researchers further proposed a deep learning model combining biological vision. For example, Tang et al. [20] proposed a biologically inspired model for contour detection by simulating nCRF modulation using deep learning techniques, and achieved good performance. Lin et al. [21] simulated the information processing transfer mechanism of retina/LGN to design the pre-enhanced network, and achieved high-performance extraction of image edges by combining the encoding network-decoding network. Fan et al. [22] proposed a convolutional neural network for facial expression recognition, and achieved performance improvement by using knowledge transfer learning (KTL) to simulate the cognitive learning ability of humans. Figure 1 shows the process and connection between the biological visual system and neural network in processing visual information.

Moreover, transformers have also attracted the attention of computer vision researchers [25,26,27]. The Swin Transformer [27] achieved the best performance in multiple computer vision tasks, breaking the dominance of CNN in computer vision tasks. Later, some researchers were inspired to combine biological vision with transformers, and proposed an edge detection model that simulates visual pathways [28] and an edge detection network that simulates the selective mechanism of the visual cortex [29], which have achieved good performance. This also provides a good theoretical basis and direction for our research.

In this paper, inspired by the selective mechanism in the visual cortex, we propose a Bio-inspired network (BiNet) for ultrasonic image classification of fatty liver. First, we use the attention mechanism to simulate the selection mechanism of the visual cortex and carry out step-by-step processing and feature extraction on the input image to achieve the extraction of the region of interest in the fatty liver ultrasound image. Secondly, according to the inhibitory effect of the nCRF response of primary visual cortex neurons on the CRF response, a receptive field feature extraction module is designed to extract texture features in input images, suppress useless background information, and enhance the feature extraction ability of the model. Finally, we use the full connection layer to classify the extracted features and output the final prediction results. In addition, we change the previous method of extracting features using the attention mechanism, and design new parallel attention blocks to achieve better performance by integrating more feature information. The contributions of this paper are summarized as follows:

1. A Bio-inspired network (BiNet) for liver ultrasound image classification is presented by simulating the selective mechanism and feedback regulation mechanism of the ventral pathway visual cortex using a self-attention mechanism, and realized the extraction of important features in ultrasound images. In addition, a receptive field feature extraction module is designed based on the inhibition characteristics of the V1 neuron nCRF response to the CRF response, which further improves the accuracy of liver ultrasound image classification;
2. A new parallel attention module is proposed. Unlike the previous attention methods that process input features sequentially, the parallel attention block has the same input. The input features are processed by two different attention paths at the same time, after which the outputs of both are fused and passed to the next stage as the input. By integrating more characteristic information, the module makes different information fully integrated and improves the overall performance of the model;
3. A new dataset for fatty liver ultrasound image classification is constructed to train, validate, and test the proposed method. A total of 250 liver ultrasound images are collected in the new dataset, including 100 normal liver ultrasound images and 150 abnormal liver ultrasound images.

The rest of the paper is organized as follows: In Section 2, we describe the proposed method in detail. In Section 3, we present the results of the proposed method on different datasets and compare them with other methods. In Section 4 and Section 5, we discuss and summarize this work.

## 2. Materials and Methods

### 2.1. Datasets

**Figure 2:**
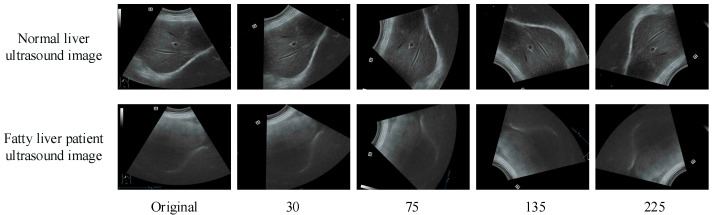
From left to right are the original image, rotated by 30°, 75°, 135°, and 225°.

In this section, we test the proposed method on two different datasets. The first dataset is proposed by Byra et al. [17], which was collected from 55 participants with 550 images in total. The other dataset is a self-built database, which was collected from elderly medical examination patients over 65 years of age who visited the Tiaodenghe Community Health Service Center in the Chenghua District of Chengdu between 2020 and 2022. A total of 250 images were selected from the 1265 participants after excluding images that were ambiguous due to large liver area occupancy, gas interference, and obesity. They included 100 ultrasound images of normal livers and 150 ultrasound images of moderately severe fatty livers. The diagnosis was reviewed and confirmed by two doctors. The images are 3-channel RGB with 8-bit depth per channel and a size of 720 × 480. We then divided the training verification set and the test set according to the ratio of 4:1. To better train the proposed model, we carried out data enhancement on the training set and verification set by randomly scaling, flipping, and rotating different angles, and finally formed a new amplification dataset. Figure 2 shows ultrasound images of the normal and the fatty liver patients randomly selected from our dataset, as well as the results after rotation at different angles.

### 2.2. Selective Mechanisms of the Visual Cortex in the Biological Visual System

It has been shown that the visual cortex is an integral part of the biological visual system for processing visual information. The visual cortex can be divided into the “ventral pathway” and “dorsal pathway” according to the direction in which visual information is processed and transmitted. Among them, the transmission process from V1→V2→V4→IT is called the “ventral pathway”, which mainly deals with color, shape, and direction information used for object shape recognition and classification in visual information. The processing and transmission process from V1→V2→V3→MT is called “dorsal pathway”, which is mainly used for the analysis of moving objects [30,31,32]. In this paper, we perform feature extraction and classification on ultrasound images of fatty livers, so we need to focus on the ventral pathway, which is more important for object recognition and classification. The blue and green arrows in Figure 1 indicate the direction of information transmission in the ventral and dorsal pathways, respectively.

With the continuous exploration of researchers, some experts and scholars [33,34,35,36] found the selective mechanism in the visual cortex, and inspired by this, proposed a widely used attention mechanism. Studies in neuroscience have also shown that selective mechanisms exist in V1, V2, and V4 of the biological visual pathway when processing visual information. That is, they respond differently to different information, and pay more attention to some sensitive and important information, while ignoring some details that are considered unimportant. In addition to the selective attention mechanism, in biological visual systems, receptive fields are areas of neurons that vigorously respond to optimal stimuli. The CRF is the area of the cell that responds to bars or edges of optimal size and orientation. When a cell is activated by a stimulus in its CRF, another stimulus that occurs simultaneously outside the region will have an inhibitory effect on the cell response, and the part that has an effect outside the region is called the nCRF. The receptive field regulation mechanism of neurons in the V1 region can effectively suppress background textures in the image, which is beneficial for efficient feature extraction [20,23]. As a high-level cortical region in the biological visual pathway, the IT region plays an important role in object recognition, classification, and feature integration [37,38]. It has been shown that when the IT area is damaged, it directly affects the brain’s ability to recognize objects [38]. Inspired by the study, we design a BiNet algorithm for liver ultrasound image classification by simulating the selection mechanism and feedback regulation mechanism of the visual cortex. The algorithm can selectively extract the regions of interest in the ultrasound image according to the global information of the image. Subsequently, accurate classification of fatty liver ultrasound images is achieved by using classification blocks to simulate the function of IT layers to integrate information based on the connection between neural networks and biological vision, BiNet models V1, V2, V4, and IT in the ventral pathway, and forms a reasonable correspondence with them in terms of function and structure.

### 2.3. Overall Network Structure

**Figure 3:**
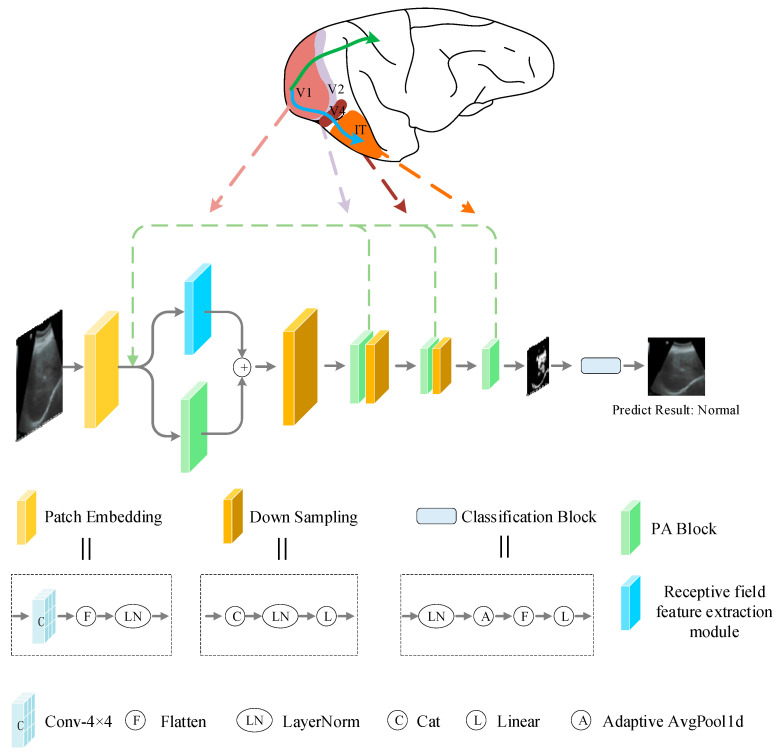
Overall structure diagram of BiNet. Receptive field feature extraction module is described in detail in Figure 4. PA block is a parallel attention block proposed in this paper, which is described in detail in Section 2.5.

Figure 3 shows the overall structure diagram of the Bio-inspired network (BiNet) proposed in this paper. It mainly includes two parts: feature extraction and classification. The feature extraction part performs the step-by-step extraction of feature information by superimposing a parallel attention block (PA block) and down-sampling module (DS). Using the down-sampling module as the boundary, the feature extraction part is divided into three stages, which respectively correspond to V1, V2, and V4 regions in the biological vision system. The first stage corresponds to V1, and includes a patch embedding (PE) operation, a parallel attention block, a receptive field feature extraction module (RFFE), and a down-sampling module to realize the preliminary processing of information. Among them, RFFE simulates the inhibition characteristics of the nCRF to CRF response in the primary visual cortex region V1, realizes the inhibition of background texture in the image, and enhances the feature extraction capability of the model in the first stage, which is described in detail in Section 2.4. The second stage corresponds to V2, and contains a parallel attention block and down-sampling module, which realizes the further processing of the information in the first stage. The third stage corresponds to V4, which contains two parallel attention blocks and down-sampling modules. Through processing the information of the first two stages, more advanced characteristic information is obtained. After that, the feedback adjustment mechanism of the higher visual cortex to the primary visual cortex in the visual system was simulated to establish the feedback connection. Finally, the feature information processed step by step was passed to the classification block, which was then processed by the LN layer, the global average pooling layer, and the fully connected layer to output the final prediction results. The classification block corresponds to the IT layer in the biological vision system, and achieves the integration function of feature information. In BiNet, the parallel attention block simulates the selective mechanism of the visual cortex in the biological vision system, which can selectively extract important features from the global information, while achieving the level-by-level extraction of image features. Specific implementation is as follows:

(1) $$F_{0}=PE\left(I\right)+{US}_{i-1}\left(F_{i}\right)+{US}_{3}\left(F_{4}\right),$$

(2) $$F_{1}=DS\left(PA\left(F_{0}\right)+RFFE\left(F_{0}\right)\right),$$

(3) $$F_{i}=DS\left(PA\left(F_{i-1}\right)\right),$$

(4) $$l=CB\left(F_{4}\right),$$

where $I\in R^{3\times H\times W}$ represents the input liver ultrasound image (H and W denote height and width). $F_{0}\in R^{2\left(C\times 4\times 4\right)\times \frac{H}{4}\times \frac{W}{4}}$, $F_{i}\in R^{2^{\left(i+1\right)}\left(C\times 4\times 4\right)\times \frac{H}{2^{\left(i+2\right)}}\times \frac{W}{2^{\left(i+2\right)}}}$, C=3, i∈2,3, $F_{4}=PA\left(F_{3}\right)$. l represents the final classification result. PE is a patch embedding operation that implements a transformation process of token information by mapping each patch information to a high-dimensional space. The detailed process is $PE=LN\left(Flatten\left(Conv2D\left(I\right)\right)\right)$ LN represents the Layer Normalization operation used. Flatten indicates flattening, converting multi-dimensional data into one-dimensional data. Conv2D represents a two-dimensional convolution operation. ${US}_{i-1}$ represents the up-sampling operation, and i − 1 represents the number of up-sampling. PA is a parallel attention block; the specific operation is shown in Equations (9)–(13). In the formula, DS and CB can be expressed as $DS=Linear\left(LN\left(Cat\left(F_{i}\right)\right)\right)$, Cat indicates a concatenation operation, where i∈2,3, $CB=Linear\left(Flatten\left(Adaptiveaveragepooling1d\left(LN\left(F_{i}\right)\right)\right)\right)$, i=4, Linear indicates the fully connected layer. DS implements a patch merging operation in addition to down-sampling the feature map [27].

### 2.4. Receptive Field Feature Extraction Module

**Figure 4:**
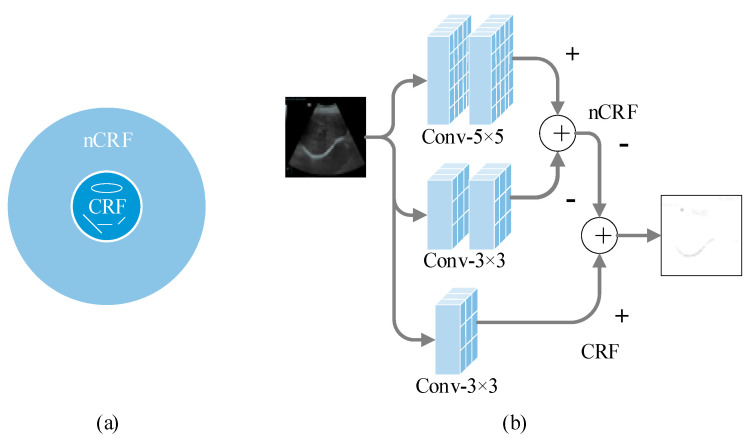
(**a**) is the range of CRF and nCRF. (**b**) is a detailed structure diagram of the receptive field feature extraction module.

The receptive field of V1 neurons in the biological vision system shows the inhibition of the periphery to the center, that is, the inhibition of the nCRF response to the CRF response. When extracting feature information, this inhibition is manifested as the inhibition of background texture, which is helpful to extract lines and useful features in the image. Figure 4a shows the scope of action of CRF and nCRF. Some researchers [20,23] have realized the extraction of effective features such as object contour by simulating this characteristic of the receptive field of neurons in the V1 region. Tang et al. [20] proposed a new biomimetic model by combining deep learning with the inhibition of CRF responses by nCRF responses. Inspired by this, we use the attention mechanism to simulate V1 selectivity in the first stage. Meanwhile, the receptive field feature extraction module is designed to simulate the inhibition effect of the nCRF response on the CRF response, which enhances the feature extraction capability of the first stage, and improves the performance of the model. The detailed construction is shown in Figure 4. The input image is processed by two 5 × 5 and two 3 × 3 convolution layers, and fused to obtain the result of the simulated nCRF response. The input image is also convolved with a 3 × 3 map to obtain the simulating CRF response. Then, the result of the nCRF response is removed from the result of the CRF response, and the result after the inhibition of the CRF response is obtained. The “−” represents the result to be suppressed, and “+” represents the response before inhibition.

The following formula is the specific operation of Figure 4a,b. Equation (5) is the suppression term of the CRF response and nCRF by the difference of Gaussian (DOG) simulation in traditional methods. More is explained in [23]. Equations (6)–(8) are specific operations of simulating the non-classical receptive field response and suppressing the classical receptive field response.

(5) $${DoG}_{\sigma }\left(x,y\right)=\frac{1}{2\pi {\left(4\sigma \right)}^{2}}e^{-\frac{x^{2}+y^{2}}{2{\left(4\sigma \right)}^{2}}}-\frac{1}{2\pi \sigma^{2}}e^{-\frac{x^{2}+y^{2}}{2\sigma^{2}}},$$

(6) $$nCRF=C_{5\times 5}\left(C_{5\times 5}\left(I\right)\right)-C_{3\times 3}\left(C_{3\times 3}\left(I\right)\right),$$

(7) $$CRF=C_{3\times 3}\left(I\right)$$

(8) Output=CRF−nCRF

where $I\in R^{3\times H\times W}$ represents the input liver ultrasound image (H and W denote height and width). $C_{m\times n}$ is the convolution, m and n are the size of the convolution kernel, m,n∈1,3,5.

### 2.5. Parallel Attention Block

The Swin Transformer [27] addresses the high complexity of the previous transformer layer [39] by introducing self-attention mechanisms and local window movement. Moreover, in Swin, if the input image $I\in R^{3\times H\times W}$ is given, Swin first divides the input into multiple non-overlapping S × S local windows, and then calculates the concern of feature F in each S × S window. Relevant parameters are calculated as follows:

(9) $$Q=W_{q}F,K=W_{k}F,V=W_{v}F,$$

where $W_{q}$, $W_{k}$, and $W_{v}$ represent different mapping matrices. Q, K, and V to calculate the self-attention matrix:

(10) $$Attention\left(Q,K,V\right)=softmax\left(\frac{QK^{T}}{\sqrt{d}}+b\right)V,$$

where b is the position deviation that can be learned; since the initial transformer layer computes self-attention multiple times in parallel, it is called multi-head self-attention (MSA). By combining with multi-layer perceptron (MLP), MSA can better extract the feature information of each window. In Swin [27], MSA is changed to Window Multi-head Self-Attention (WMSA) and Shift Window Multi-head Self-Attention (SWMSA). The input images are first processed by WMSA to calculate the attention within a window, and later by SWMSA to calculate the attention between different windows. In order to establish long-term relationships of feature information, WMSA and SWMSA can be used interchangeably when constructing the network.

**Figure 5:**
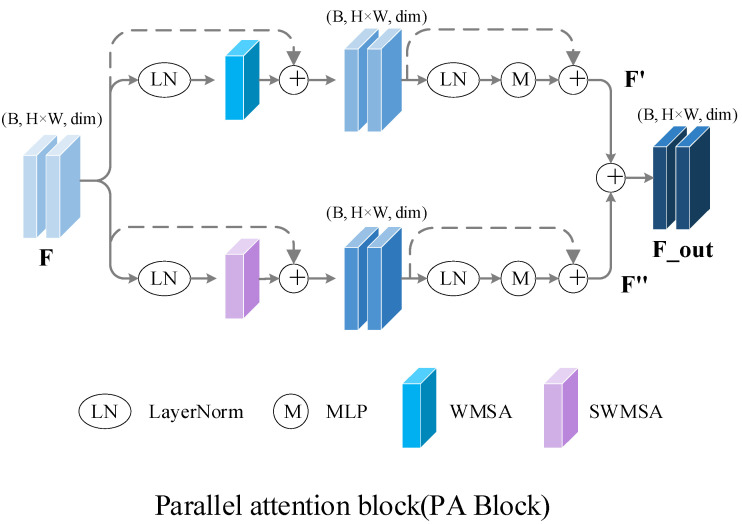
Detailed structure of parallel attention block. The input features are processed by two different attention paths, where one attention path only computes the attention within a window and no information is exchanged between different windows. The other attention path fuses the information between the different windows. The output of the last two attention paths is fused as the input for the next stage.

Recently, Swin has achieved the best results in some visual tasks with its hierarchical design and extraction of global features. The proposed WMSA and SWMSA also show strong and effective feature extraction ability when dealing with global features. However, the diagnosis of hepatic steatosis by ultrasonic images requires the extraction of distinct features from ultrasonic images. This is also the key problem to improve the diagnostic accuracy of hepatic steatosis. Therefore, inspired by biological vision mechanisms, we use attention mechanisms to simulate selective neural mechanisms in ventral pathways to design parallel attention blocks that can simultaneously process input images. Its structure is shown in Figure 5. In the parallel attention module, the input images are processed by WMSA and SWMSA, respectively, and the two do not interfere with each other during processing, after which the outputs of the two are fused after a series of calculations and residual connections to achieve the extraction and fusion of different feature information. In addition, WMSA and SWMSA simulate the selective mechanism of the visual cortex in the biological visual system, and realize the extraction of globally effective features by processing input images. The specific calculations are as follows:

(11) $$F^{\prime }=MLP\left(LN\left(WMSA\left(LN\left(F\right)\right)+F\right)\right)+\left(WMSA\left(LN\left(F\right)\right)+F\right),$$

(12) $$F^{\prime \prime }=MLP\left(LN\left(SWMSA\left(LN\left(F\right)\right)+F\right)\right)+\left(SWMSA\left(LN\left(F\right)\right)+F\right),$$

(13) $$F_{out}=F^{\prime }+F^{\prime \prime },$$

MLP and LN are represented as follows:

(14) $$MLP=Linear\left(GELU\left(Linear\left(x\right)\right)\right),$$

(15) $$y=\frac{x-E\left[x\right]}{\sqrt{Var\left[x\right]+\epsilon }}\times \gamma +\beta ,$$

where ϵ is a small constant such that $Var\left[x\right]+\epsilon >0$. γ is the gain and β is the bias, and the combination keeps the information from being corrupted. More details are given in [40].

### 2.6. Implementation Details and Evaluation Metrics Methods

We implement our model on Pytorch. In training, we use migration learning methods to initialize the modified BiNet with parameters from the Swin Transformer pre-trained on ImageNet-1K [41]. We update the parameters using the Adam optimizer, setting the global learning rate to 0.0001, epoch to 10, and weight decay to 5 × 10^−2^. The size of the input image is 224 × 224. The device used is an NVIDIA GeForce 1080Ti GPU. For fair comparison, we use the same evaluation criteria as in the previous work [1,2,14,42,43] and calculate the accuracy, sensitivity, and specificity of the model. In addition, we also calculate the F1 score of the proposed model. The specific calculations are as follows:

(16) $$Accuracy=\frac{TP+TN}{TP+FP+TN+FN},$$

(17) $$Sensitivity=\frac{TP}{TP+FN},$$

(18) $$Specificity=\frac{TN}{TN+FP},$$

(19) $$F1-score=\frac{2\times P\times R}{P+R},$$

where TP, TN, FP, and FN represent the number of true positive, true negative, false positive, and false negative detection in the classification process, respectively. P is for precision, where $P=TP/\left(TP+FP\right)$, and it represents the proportion of true positives in true positives and false positives. R is for recall rate, also known as sensitivity, which represents the proportion of true positives in true positives and false negatives.

## 3. Results

In this section, we make a detailed experimental analysis of the proposed diagnostic method of liver steatosis on the dataset proposed in this paper and publicly available datasets.

### 3.1. Comparison of Results under Different Parameters

Aiming at the parameter setting in the training process, we conduct quantitative research and comparison on the BiNet model on the dataset. First, the results for different epochs of training and testing under the same conditions are shown in Table 1. By comparison, we can get the best performance of the model when epoch is 10. In addition, we adopt the same method to train and test the results of different learning rates under the same conditions. Table 2 shows the experimental results for different learning rates, from which we can find that the model has the best performance when the learning rate is set to 0.0001.

### 3.2. Result Verification of Parallel Attention Blocks

**Figure 6:**
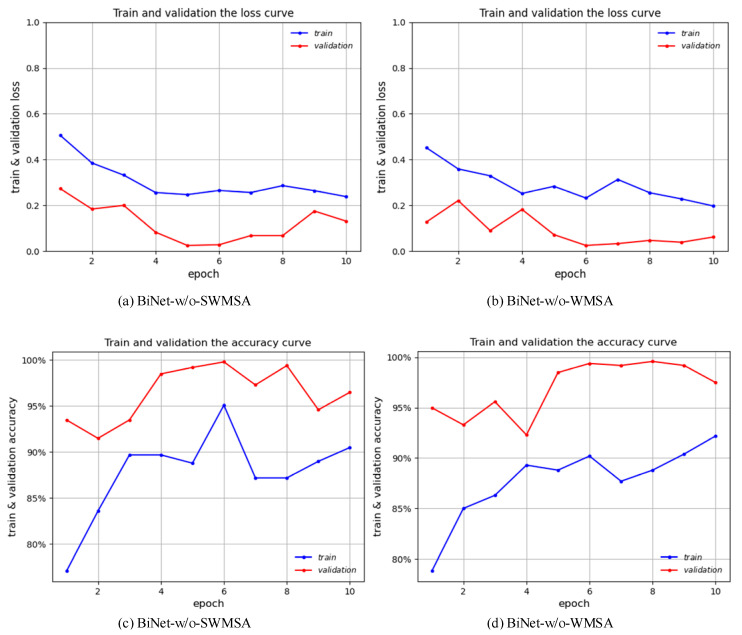
Loss change curves and accuracy change curves of BiNet-w/o-SWMSA and BiNet-w/o-WMSA on the training and validation sets.

To further validate the effectiveness of parallel attention blocks in BiNet, we conduct detailed ablation experiments on the dataset presented in this paper. First, we train and test the original Swin Transformer, after which we add the design parallel attention blocks to the backbone network, BiNet, to train and test it. In addition, to demonstrate that the parallel attention block can adequately fuse the outputs of the two attention paths, we also test the results when there is only one attention path in BiNet separately. That is, the results when only WSMA and only SWMSA are used. BiNet-w/o-SWMSA indicates that only WMSA is used, and BiNet-w/o-WMSA indicates that only SWMSA is used. All experimental results are shown in Table 3, and the training process of BiNet-w/o-SWMSA and BiNet-w/o-WMSA is shown in Figure 6. As can be seen from the experimental results in Table 3, BiNet achieves the best results on both the validation and test sets after using the parallel attention block, and outperforms the other models by 2–4% in accuracy. This also indicates that the parallel attention block proposed in this paper is more competitive than the original processing method, and can achieve more accurate liver ultrasound image classification.

### 3.3. Comparison with Other Models

**Figure 7:**
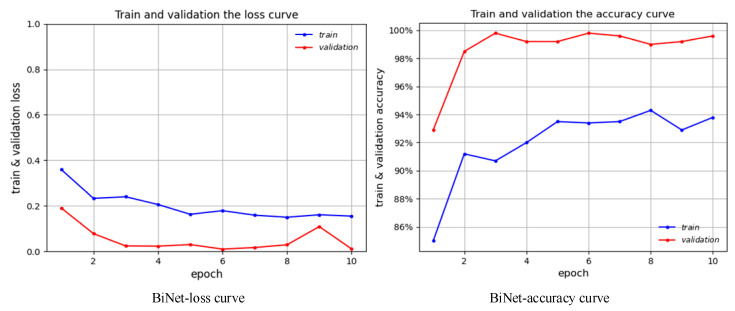
Change curves of loss and accuracy of BiNet.

**Figure 8:**
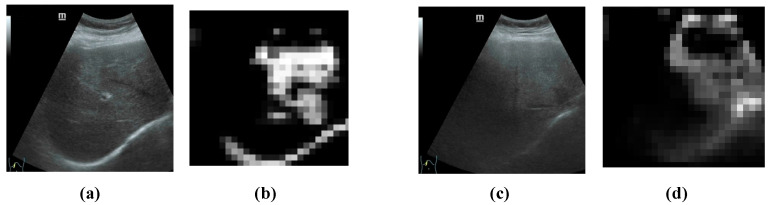
BiNet feature extraction map. From left to right, (**a**) normal liver ultrasound image, (**b**) feature map extracted from normal liver ultrasound image, (**c**) fatty liver patient ultrasound image, and (**d**) feature map extracted from fatty liver patient ultrasound image.

As shown in Figure 7, we train and verify BiNet’s loss curve and accuracy curve on the amplified data set. In addition, we also conduct a detailed evaluation of the training model on the test set. Table 4 compares our proposed method, BiNet, with other diagnostic methods for hepatic steatosis. Figure 8 shows the results before and after BiNet processing.

As can be seen from Figure 7, BiNet gradually decreases the loss value and increases the accuracy rate during the training process without large fluctuations, and the model gradually converges and achieves a better performance. In addition, it can be seen from Table 4 that BiNet has achieved good results among all the methods, and its accuracy, sensitivity, and specificity are all higher than other methods. This further demonstrates that our method is highly competitive among all diagnostic methods for steatosis.

## 4. Discussion

As we mentioned in the introduction, hepatic steatosis diagnosis has important implications for preventing liver disease and maintaining human health. However, the ultrasound images widely used in the diagnosis of hepatic steatosis usually have problems, such as low quality, noise interference, and dependence on the doctor’s experience, which affect the accuracy of the diagnosis of hepatic steatosis. To this end, some researchers have proposed ways to improve image quality, build complex models, and use machine learning methods to address these problems. These methods overcome the problems in the diagnosis of hepatic steatosis to a certain extent, and improve diagnostic accuracy, but there are still some limitations. After that, the convolutional neural network has been widely used in various fields because of its excellent performance in various visual tasks and image processing tasks. The diagnostic method of liver steatosis based on the convolutional neural network has also been proposed by researchers and achieved high accuracy. However, with the gradual deepening of research, some researchers found that it is difficult to improve the performance of the model only by relying on experience and a large number of experiments, and it usually leads to complex models, low efficiency, and occupying a lot of computing resources. In recent years, the process and physiological mechanism of visual information processing in biological visual systems have received much attention from researchers. Based on the physiological mechanisms in biological vision systems, some researchers have proposed methods combined with deep learning to achieve good results in various computer vision tasks.

Inspired by the ventral pathway in the biological vision system, we design the BiNet for the diagnosis of fatty degeneration in liver ultrasound images by combining the receptive field regulation mechanism of neurons in the V1 region. Moreover, the selectivity mechanism of V1, V2, and V4 regions, and the feedback regulation mechanism of higher cortical regions to lower cortical regions are designed and implemented. The model performance is verified by experiments on datasets. However, our approach has certain limitations. In this work, we mainly focus on the function of the ventral pathway in the biological visual system and its physiological mechanism. However, in the real biological visual system, the processing and transmission of visual information start from the photoreceptors, and the visual information goes through a series of processing steps before being transmitted to the V1 region. Moreover, BiNet is only trained and tested on two datasets, and its scalability is somewhat limited. The BiNet presented here mimics the physiological mechanisms of the visual cortex in the ventral pathway and the processing of visual information in the ventral pathway, making its structural design more interpretable. In this paper, instead of using only deep learning or biological vision, we model the selection mechanism of the visual cortex using the attention mechanism. This provides a new direction for further research and promotes the integration of biological vision and computer vision. In future work, we may also incorporate more effective biological vision mechanisms into deep learning methods to improve the overall performance of the model. The classification performance will be improved as more datasets become available.

## 5. Conclusions

In this paper, we propose a biologically inspired network for the diagnosis of hepatic steatosis by simulating the selection and feedback regulation mechanisms of the visual cortex in biological visual systems. Different from the previous CNN-based method, BiNet can not only extract simple features in liver ultrasound images, but also selectively extract areas of interest in ultrasound images through the attention mechanism to achieve accurate image classification. We conducted detailed experiments and evaluations of BiNet on the dataset, and the results showed that BiNet achieved optimal performance with accuracy, sensitivity, and specificity of 98.0%, 100%, and 96.0%, respectively. It can also be seen that the model proposed in this paper is competitive among all methods, which is conducive to reducing the pressure on doctors in clinical practice and reducing the occupation and consumption of resources. Moreover, in this paper, instead of using only deep learning or biological vision, we model the selection mechanism of the visual cortex using the attention mechanism. This provides a new direction for further research and promotes the integration of biological vision and computer vision. In future work, we may also consider incorporating more effective biological vision mechanisms into deep learning methods to improve the overall performance of the model.

## Figures and Tables

**Table 1:** Comparison of results of different training epochs.

Method	Epoch	Accuracy (Validation)	Accuracy (Test)	Sensitivity	Specificity	F1-Score
BiNet	5	94.0%	96.0%	100.0%	92.0%	0.96
BiNet	8	98.5%	96.0%	100.0%	92.0%	0.96
BiNet	10	99.8%	98.0%	100.0%	96.0%	0.98

**Table 2:** Comparison of results of different learning rates.

Method	Lr	Accuracy (Validation)	Accuracy (Test)	Sensitivity	Specificity	F1-Score
BiNet	0.001	81.5%	82.0%	64.0%	100.0%	0.78
BiNet	0.00001	98.3%	90.0%	80.0%	100.0%	0.89
BiNet	0.0001	99.8%	98.0%	100.0%	96.0%	0.98

**Table 3:** Effectiveness of parallel attention blocks in BiNet.

Method	Accuracy (Validation)	Accuracy (Test)	Sensitivity	Specificity	F1-score
Swin_original	99.4%	96.0%	92.0%	100.0%	0.96
**BiNet**	99.8%	98.0%	100.0%	96.0%	0.98
BiNet-w/o-SWMSA	99.8%	96.0%	92.0%	100.0%	0.96
BiNet-w/o-WMSA	99.6%	98.0%	96.0%	100.0%	0.98

**Table 4:** Comparison of BiNet with other methods. † indicates the results of the reference.

Authors	Dataset	Accuracy	Sensitivity	Specificity	F1-Score
Acharya et al. [44]	Private	93.3% †	-	-	-
Sharma et al. [45]	Delta Diagnostic Centre Patiala, India, Private	95.55% †	-	-	-
Andrea et al. [46]	Coimbra University Hospital, Private	kNN:74.05% † ANN:76.92% † SVM: 79.77% †	-	-	-
Gaber et al. [42]	Private	95.71% †	97.05% †	94.44% †	0.956
Zhang et al. [14]	Private	90.0% †	81.0% †	92.0% †	-
Byra et al. [17]	Medical University of Warsaw, Poland, Publicly available	96.3% †	100.0% †	88.20% †	-
BiNet (ours)	Medical University of Warsaw, Poland, Publicly available	99.1%	100.0%	98.7%	0.986
BiNet (ours)	Private	98.0%	100.0%	96.0%	0.980

## Data Availability

Not applicable.
